# Feasibility of Enzymatic Protein Extraction from a Dehydrated Fish Biomass Obtained from Unsorted Canned Yellowfin Tuna Side Streams: Part I

**DOI:** 10.3390/gels9090760

**Published:** 2023-09-18

**Authors:** Federica Grasso, Diego Méndez-Paz, Rebeca Vázquez Sobrado, Valentina Orlandi, Federica Turrini, Giulia De Negri Atanasio, Elena Grasselli, Micaela Tiso, Raffaella Boggia

**Affiliations:** 1 Department of Pharmacy, University of Genova, Viale Cembrano 4, 16148 Genova, Italy; federica.grasso@edu.unige.it (F.G.); valentina.orlandi@edu.unige.it (V.O.); raffaella.boggia@unige.it (R.B.); 2 ANFACO-CECOPESCA, Department of Circular Economy, Colexio Universitario, 36310 Vigo, Spain; dmendez@anfaco.es (D.M.-P.); rebeca.vazquez@anfaco.es (R.V.S.); 3 National Center for the Development of New Technologies in Agriculture (Agritech), 80121 Napoli, Italy; elena.grasselli@unige.it; 4 Department of Earth, Environmental and Life Sciences, University of Genova, Corso Europa 26, 16132 Genova, Italy; giulia.denegriatanasio@edu.unige.it; 5 MICAMO LAB, Via XX Settembre 33/10, 16121 Genova, Italy; m.tiso@micamo.com; 6 National Biodiversity Future Center (NBFC), 90133 Palermo, Italy

**Keywords:** unsorted fish by-products, tuna side stream, fish proteins, gelatin, non-collagenous proteins, circular economy, enzymatic extraction, rheological properties

## Abstract

This study presents for the first time a scalable process for the extraction of valuable proteins starting from samples of unsorted mixed tuna scraps which were previously dehydrated by an industrial patented process. The aims of this work were both to avoid the onerous sorting step of tuna leftovers, which generally consists of isolating skin and bones for collagen/gelatin extraction, and to improve the logistic of managing highly perishable biomass thanks to the reduction in its volume and to its microbiological stabilization. In view of a zero-waste economy, all the protein fractions (namely, non-collagenous proteins NCs and ALKs, gelatin, and hydrolyzed gelatin peptides, HGPs) isolated in the proposed single cascade flowchart were stabilized and preliminarily characterized. The extraction flowchart proposed allows one to obtain the following most promising compounds: 1.7 g of gelatin, 3.2 g of HGPs, and 14.6 g of NCs per 100 g of dehydrated starting material. A focus on oven-dried gelatin was reported in terms of proximate analysis, amino acid composition, color parameters, FT-IR spectrum, pH, and viscoelastic properties (5 mPa·s of viscosity and 14.3 °C of gelling temperature). All the obtained extracts are intended to be exploited in food supplements, feed, fertilizers/plant bio-stimulants, packaging, and the cosmetic industry.

## 1. Introduction

According to the Food and Agriculture Organization (FAO), fish production has been growing at a 3% yearly rate since 1961, reaching 214 million tons of fish produced in 2020 [1,2]. The amount of fish waste produced in recent years has therefore increased noticeably and it is believed that almost two-thirds of that is discarded, raising serious economic and environmental issues [3].

One of the main goals of EcoeFISHent, a project of the Horizon 2020 Program–Green Deal (G.A. ID 101036428) [4], is represented by the enhancement of discarded and underused biomass deriving from fish supply chain for the sustainable extraction of high-value bioactive molecules of nutraceutical and cosmetic interest, following the “zero-waste” approach in accordance with the 12th Sustainable Development Goal (SDG) [5]. One of the major problems for the valorization of the side streams of the fish supply chain is precisely the treatment of non-separated biomass, not only the fish filleting processing residues but also the by-catches and/or unwanted catches (such as undersized fish). For this reason, fish side streams are often only partially valorized and used in low-value products such as animal feed and fertilizers [6]. Moreover, fish represents a highly perishable biomass, and its potential exploitation is strongly connected to the microbiological activity that has to be minimized by reducing the presence of water, which constitutes about 50–80% of these fish side streams [7]. In all these cases, starting from stabilized biomass (for example, through industrial dehydration) could represent a crucial advantage to avoid biomass waste.

The aim of this study is therefore the valorization of dehydrated unsorted tuna side streams, called “EcoeFISHent biomass (YDFB, Yes Dehydrated Fish Biomass)”, coming from the tuna canning industry. The exploitation of these fish leftovers takes place with a scalable process of extraction of high-value proteins, such as gelatin, with future applications in multiple sectors, and Hydrolyzed Gelatin Peptides (HGPs), with a steadily increasing interest linked to their biological activity [8], but also protein hydrolysates, such as non-collagenous proteins rich in essential nutrients and bioactive components, of growing interest both for the food supplements market and the enriched foods one [9].

Tuna represents one of the most commercialized and consumed fish in the EU, in particular as a canned product [10], with increasing demand all over the world. The tuna industry generates a big amount of by-products, including heads, viscera, bones, and skin, since the white meat is the only part used in canning or sashimi [11]. Tuna side streams, corresponding to 55–70% of the total fish weight [12], need to be exploited since it is a precious source of high value bioactive compounds, i.e., hydrolysates, gelatin, collagen, and PUFAs [13].

Gelatin is a water-soluble biopolymer, usually obtained by denaturation of collagen, which is present only in animals. In particular, commercial gelatin mostly derives from bovine and porcine discards (bones, skin, cartilage, and tendons) [14].

Nowadays, the demand for gelatin of fish origin is increasing, because, unlike the bovine type, marine gelatin does not present problematic limitations linked to infectious diseases such as Bovine Spongiform Encephalopathy (BSE) [15], Foot and Mouth Disease (FMD), or religious constraints that do not allow its consumption such as for Hinduism, Islam, and Judaism [16]. Fish gelatin is approved by all these communities and, for this reason, it is considered a valid alternative to the terrestrial ones; nevertheless, it has to be considered that fish gelatin usually contains less quantity of typical amino acids like proline and hydroxyproline [17] compared to mammalian gelatin, with consequences for physicochemical and functional properties such as gel strength, viscosity, and melting point [18]. In addition to the type of starting material, extractive conditions can affect the properties of gelatin too. Collagenous material, especially gelatin, is frequently used in the food, pharmaceutical, cosmetic, and packaging sectors [19]; gelatin is known to have significant technological properties including the ability of gelation and stabilization, and it is used as emulsifying agent and for encapsulation [20].

HGPs obtained from fish side streams have demonstrated several bioactive properties in the literature such as antioxidant activity, anticancer activity, and fat binding ability, proving to be potential functional ingredients with high added value for food applications [8,21]. Obtaining gelatin and HGPs from different fish sources starting from previously separated biomasses (e.g., only skin, bones) is already widespread in the literature [22]. This is performed in order to maximize the yields, since these previously isolated parts contain the majority of the collagen. However, for many industrial fish supply chains, the separation step of side streams is not affordable, making the exploitation of these leftovers difficult to realize.

Concerning gelatin extraction protocols usually reported in the literature, they are generally similar with slight differences depending on the starting biomass being treated. Typically, three main steps are performed: pretreatment to remove non-collagenous materials like lipids, globular proteins, and ashes; extraction in hot water; and the final phases of recovery and dehydration [23]. Gelatin can be categorized as type A or type B, based on the use of acid or alkali solvents, respectively. However, new extraction procedures are emerging involving the use of enzymes and/or ultrasounds to avoid/limit solvents and reduce processing time [24,25]. The quality and yield of gelatin are influenced not only by the source but also by the extraction methods and conditions employed during the process.

One of the novelties presented in this manuscript is the recovery in a single cascade flowchart of both non-collagenous proteins (hydrolysate NCs, ALKs), mainly coming from connective tissue, and collagenous compounds (gelatin and Hydrolyzed Gelatin Peptides, HGPs), derived mainly from bones and scales. Thus, this study represents a proof of concept for the EcoeFISHent project, underlining the importance of valorizing dehydrated unsorted mixed side streams coming from the tuna canning industry. The dehydration process allows for the simplification of the handling of a large amount of fish discards, allowing the extraction process to potentially start from the production one.

**Figure 1:**
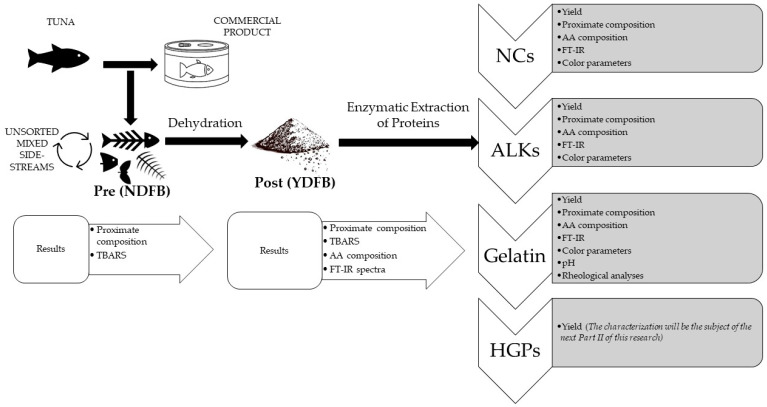
Graphical scheme of the extraction protocol. The enzymatic extraction details are reported in Figure 2.

In this first paper (Part I), the extraction flowchart of the abovementioned bioactive compounds is presented, together with a preliminary characterization of both gelatin and non-collagenous proteins (NCs, ALKs). In Part II, a more detailed focus on the collagenous extracts will be discussed in term of rheological properties and biological activity. Figure 1 shows the global scheme of the present work.

## 2. Results and Discussion

### 2.1. Proximate Composition and Monitoring of Lipid Oxidation of Samples Pre (NDFB) and Post (YDFB) Industrial Dehydration

Industrial samples of raw Yellowfin tuna’s unsorted and mixed byproducts, mainly composed of crude material of tuna, in particular heads, fins, bones, and tails, were considered both pre (NDFB, Not Dehydrated Fish Biomass) and post (YDFB, Yes Dehydrated Fish Biomass) a patented industrial dehydration treatment developed by Themis S.p.A. This process not only provides economic benefits associated with the disposal of tuna scraps volume, but also allows one to obtain a fish powder (YDFB) with a very low residual humidity that is stable over time at room temperature. The traditional methods of dehydration of fish by-products provide for a sorting step and the use of high temperatures that allow one to obtain fish meals, low-value commercial products intended as a protein source for animal feed [22].

First, proximate analysis was performed to evaluate the nutritional composition of both samples, NDFB and YDFB, namely, pre and post industrial dehydration (Table 1).

As highlighted by the results reported in Table 1, starting from biomasses with about 65% humidity (NDFB), it was possible to reach very low residual moisture values (below 5%, YDFB). This demonstrates how effectively the initial industrial dehydration process worked to stabilize this highly perishable biomass. The evaluation of the crude protein fraction has shown a high percentage of total nitrogen, around 50%, as expected for large-sized fish [26]. For this reason, YDFB can be considered an excellent candidate for the extraction of proteins, such as NCs and gelatin. As for the ashes, they have been shown to represent about 30% of the total composition, which may be linked to a high presence of bones in the biomass. Since bones are made of 70% inorganic material and the remaining 30% of collagen proteins, a high bone content may confirm that YDFB is suitable for collagenous protein extraction [27]. The lipid fraction of YDFB presented a value of 13% and could be considered for future studies in order to estimate its quality and *omega-3* composition.

Since lipid oxidation is one of the most dangerous modifications in such an unsaturated perishable matrix and could also affect the quality of the protein fraction, it was monitored in both NDFB and YDFB samples to evaluate the impact of the industrial dehydration evaluating both the primary and the secondary oxidation products. The TBARS (Thiobarbituric-Acid-Reactive Substances) test was applied, obtaining 5.6 ± 1.1 and 6.4 ± 1.8 mmol MDA/kg of fish biomass, respectively, for NDFB and YDFB, implying that the dehydration pre-treatment has not significantly influenced the lipid oxidation. In addition, the lipid oxidation was tested performing the analysis of the Peroxide Value (PV) for NDFB and YDFB, obtaining 1.0 mEq 02/kg for pre-dehydration samples and a little increment to 3.5 mEq 02/kg for the dehydrated ones.

Microbiological analyses of NDFB and YDFB were carried out to assess the performance of this specific dehydration treatment. The results demonstrate the efficiency of this dehydration treatment in stabilizing such a perishable matrix (Table 2).

The microbiological values found in both non-dehydrated samples by Themis S.p.A. technology and samples after this patented industrial dehydration treatment are typical for raw tuna. These values are compliant with ensuring food safety and the quality of raw food. Sulfite-reducing clostridia (<10 CFU/g) values indicates that the amount of sulfite-reducing clostridia and its spores in raw tuna is very low, practically undetectable. This is a positive feature, as sulfite-reducing clostridia can cause food safety issues and deterioration. The TVC value (3300 CFU/g) represents the total viable count of microorganisms in raw tuna. The value has increased compared to the sample before dehydration, probably due to differences in analysis timing. However, the data still remains within the acceptable range. Once dehydrated, the sample is expected to remain microbiologically stable. Coliforms (<10 CFU/g) and Escherichia coli β-gluc. + (<10 CFU/g) values indicate a very low presence and absence of coliforms and Escherichia coli, respectively. These parameters are important indicators of fecal contamination, and their absence or low presence is crucial for food safety. Concerning Enterobacteriaceae, the obtained value of <10 CFU/g indicates low presence or absence of Enterobacteriaceae bacteria, a group that includes potential pathogens. Staphylococci c. + at 37 °C (<10 CFU/g) value indicates low presence or absence of Staphylococcus aureus, a bacterium that can cause food poisoning. Here, too, the presence of Staphylococcus aureus should be very low or absent. Moreover, the absence of Salmonella spp. and Listeria monocytogenes, two pathogens which can cause serious illnesses, is essential for food safety. The values “Histamine <5 mg/kg” and “Total Volatile Basic Nitrogen14.9 mg/100 g” are important parameters to ensure food safety of raw tuna. Histamine can accumulate in certain types of fish, including tuna, if not properly stored. Elevated histamine levels can cause adverse reactions in consumers, known as “scombroid poisoning” or “histamine poisoning”. Histamine <5 mg/kg indicates that its content in these samples of raw tuna is very low. Acid–Base Volatile Total (ABVT) measures the amount of volatile acidic and basic substances present in the food. This value is often associated with freshness and food quality. In the context of raw tuna, a reading of 14.9 mg/100 g indicates that the tuna is likely to be fresh.

### 2.2. Characterization of Enzymatic Protein Extracts

#### 2.2.1. Extraction, Yields, and Proximate Analysis

**Figure 2:**
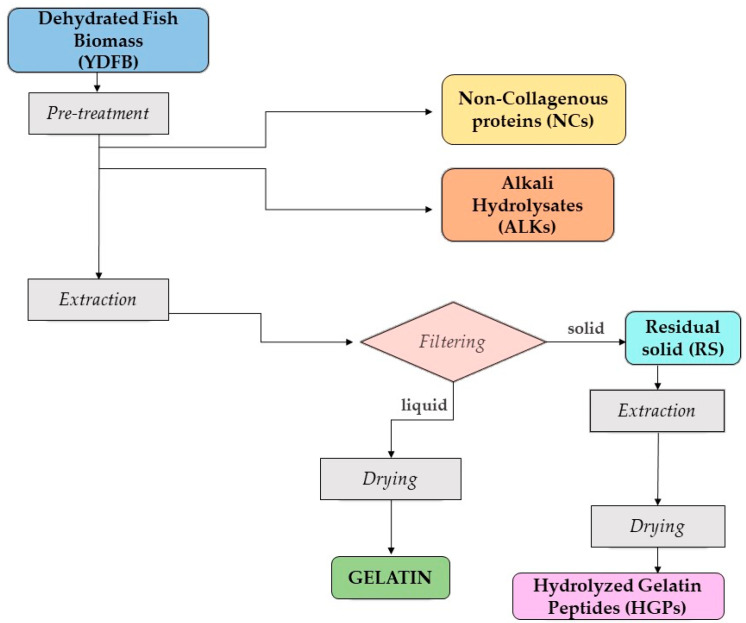
The cascade flowchart of the extraction process.

Since YDFB is constituted by a powder of dehydrated and milled unsorted tuna side streams with some residual skin and little grinded bones, the innovative extraction protocol, reported in Figure 2, aimed to recover both non-collagenous proteins (mainly from the connective tissue) and collagenous ones (mainly from skin and bones) present in this unsorted mixture. With a view of “throwing nothing away” of the starting biomass, every residue deriving from the extraction process was evaluated.

**Figure 3:**
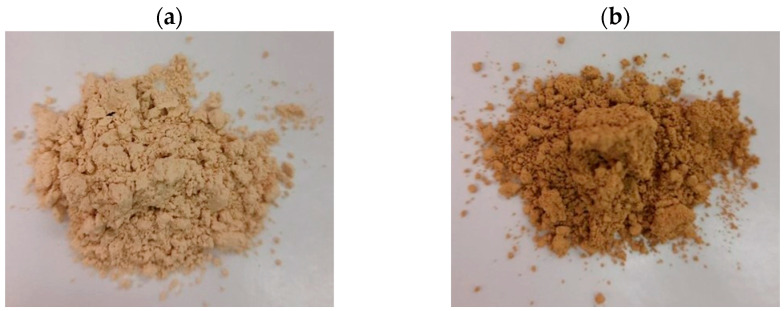
Stabilization of protein hydrolysates: (**a**) spray-dried NCs (Non-Collagenous proteins); (**b**) spray-dried ALKs (Alkali Hydrolysates).

The pH of YDFB was measured, obtaining a value of 6.10 ± 0.4. The first two steps of the extraction protocol (Figure 2), i.e., enzymatic hydrolysis and alkali pre-treatment, aimed to separate and recover two fractions of protein hydrolysates, namely, NCs and ALKs, the stabilization of which was performed by spray-drying process (Figure 3a,b).

NCs and ALKs were characterized in terms of proximate analysis and yield (Table 3). The results of the proximate analysis of NCs show a high percentage of proteins (67%), which is in keeping with the range 60–90% reported by Chalamaiah et al. [9] and makes NCs exploitable in other products (i.e., dietary food supplements). Instead, ALKs were obtained from the residual liquid fraction after alkaline treatment, mainly with the intention of removing lipids (defatting), even those bound to proteins. In fact, ALKs contain mainly lipids (about 27%) and the residual fraction of remaining non-collagenous proteins (about 33%) left over from the previous phase. ALKs composition proved that the treatment was effective for the purpose of the defatting step. Moreover, the high ash content of both NCs (24%) and ALKs (17%) could be linked to the unavoidable use of sodium hydroxide (due to the sodium content) both used in the first step to correct the pH and to remove lipids.

**Figure 4:**
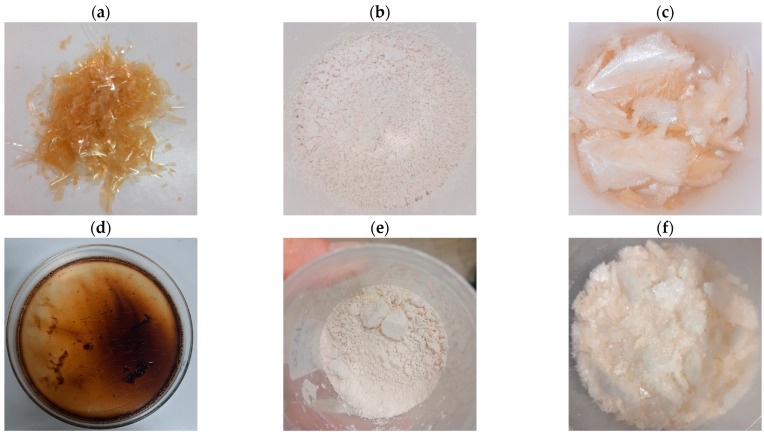
Gelatin and hydrolysate samples obtained with different dehydration processes: (**a**) oven-dried gelatin; (**b**) spray-dried gelatin (**c**) freeze-dried gelatin, (**d**) oven-dried HGPs (Hydrolyzed Gelatin Peptides), (**e**) spray-dried HGPs (Hydrolyzed Gelatin Peptides), and (**f**) freeze-dried HGPs (Hydrolyzed Gelatin Peptides).

Once the biomass was cleaned, the solid residue was subjected to further steps (see Figure 2) to isolate gelatin. The use of citric acid was effective to largely eliminate the unpleasant fishy smell. The liquid fraction containing gelatin was subjected to a drying process in an oven. This method was initially preferred over both the “spray-dryer” and the “freeze-dryer” ones, as it was considered cheaper for the possible up-scaling process. Yields of gelatin, dehydrated by oven-drying in convective mode, spray-drying, and freeze-drying (Figure 4a–c), were, respectively, as follows: 1.65 ± 0.19, 1.15 ± 0.21, and 1.70 ± 0.10 (g/100 g of starting material).

After isolation of gelatin, the residual solid was then treated with enzymatic hydrolysis to obtain HGPs (see Figure 2) stabilized by spray-drying, since oven-drying caused their deterioration. As for gelatin, the yields are reported as follows: spray-dried HGP yield was 2.43 ± 1.03 and freeze-dried HGP yield was 3.19 ± 0.40 (g/100 g of starting material) (Figure 4d–f).

A proper comparison with other scientific data is difficult to make, since gelatin is normally extracted from skin, scales, or bones, while, in this study, the authors present one of the first attempts, from what they know, at extracting gelatin and HGPs from a mixed unsorted biomass made up of dehydrated crude tuna scraps from the canning industry. In the literature, higher gelatin yields are reported; however, they were extracted from different separated starting biomasses: Qui et al. [8] obtained a 3.46% yield, starting from tuna scales; Rahman et al. [28] obtained 18% from yellowfin tuna skin; Yang et al. [29] obtained 6.37% from tuna bones (on a wet bone weight basis); Haddar et al. [30] obtained 18% from tuna head bones; and Liu et al. [31] obtained 3.9% from channel catfish head bones.

Extracted gelatins were tested for residual moisture, protein content, lipids, and ashes, and the corresponding results are reported in Table 4.

The low value of residual moisture, equal to 2%, represents a good result in terms of stability and shelf-life, and is lower than the one obtained by Baziwane et al. from grass carp skin [32]. The crude protein content of 88% reflects good purification of gelatin; in particular, the defatting step was exhaustive (lipids lower than 1%), while the demineralization could be improved. The GMIA (Gelatin Manufacturers Institute of America) [33] suggests a maximum of 2% of ashes in edible gelatin; the obtained value of 5% of ashes in this kind of sample is related to the presence of bones in YDFB, as confirmed by Muyonga et al. [34] who reported higher ash content for gelatin coming from bones compared to that extracted from skin.

#### 2.2.2. Amino Acid Analysis

In Table 5, the amino acid content of YDFB, NCs, ALKs, and gelatin is reported, and it is provided in descending order to appreciate the different composition between the starting material and the extracts. The percentage of amino acids are reported as grams of amino acid per 100 g of gelatin and as percentage of amino acids.

The aminoacidic composition may vary according to fish species, age, and size [35], as well as by fish by-product [36]. The analysis of YDFB has revealed that the major components are represented by glycine (13.80%), glutamic acid (11.92%), and alanine (7.73%), while cysteine and taurine are minor components (around 1%). Hydroxyproline and proline, representing the characteristic collagen/gelatin amino acids, contribute together with 10.57%.

Looking at the results expressed as percentage of amino acids (amino acid/100 amino acids), there are small variations between NCs and YDFB. Concerning ALKs, even if they present an amino acid composition like NCs, their content in glycine and glutamic acid are lower. However, given the mixture’s high concentration of lipids, the recovery of this protein fraction seems difficult. From the analysis of amino acids and hydroxyproline content, it is possible to notice that probably a little part of collagen was solubilized during first step of hydrolysis to remove and recovery non-collagenous proteins.

As gelatin is concerned, the aminoacidic composition is mostly defined by a high content of glycine (around 30%), a characteristic presence of proline and hydroxyproline (20%), and alanine (10%) [37]. The obtained ratio OH-Pro/Pro in gelatin, equal to 0.98, can be used to compare the result with the others reported in the literature; however, it also depends on extraction conditions [38] and it cannot be considered without taking into account all the factors involved in the extraction. The presence of amino acids and their tendency to form hydrogen bonds provides stability to the triple helix [39] and is linked to good viscoelastic properties [40] and gel strength [41].

The composition of amino acids here presented is similar to the one reported by Nurilmala et al. [42] for gelatin extracted from tuna skin (i.e., Gly 24–26%, Pro 11–12%).

#### 2.2.3. FT-IR (Fourier-Transform Infrared) Spectroscopy Analysis

**Figure 5:**
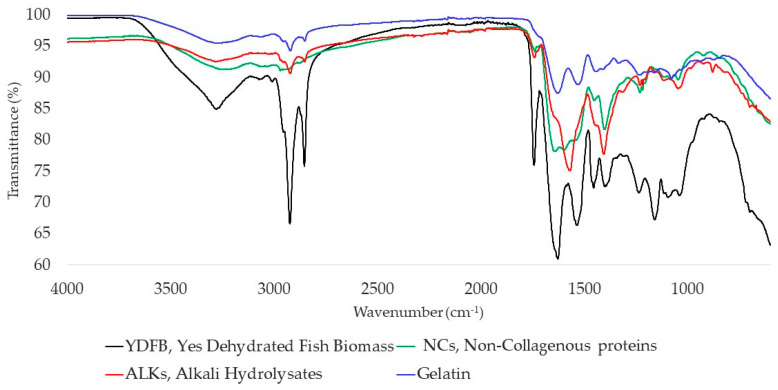
FTIR spectra of the starting material YDFB (Yes Dehydrated Fish Biomass) and obtained extracts: NCs (Non-Collagenous proteins), ALKs (Alkali Hydrolysates) and gelatin.

Fourier-Transform Infrared Spectroscopy, as a non-destructive and fast technique, was applied to extract information about the molecular structure [20], in particular, in terms of functional groups and secondary structure [43], and to evaluate the impact of hydrolysis treatment comparing the spectra of the extracts with the spectrum of YDFB (Figure 5). FT-IR spectra of spray-dried and freeze-dried gelatins are reported in Figure S1.

Nine peaks/bands (Amide A, B, I-VII) are considered representative for the identification of proteins, even if the most emblematic are Amide I and II [44].

In the spectra below, five peaks were considered as the most prominent and relevant [38]. They were registered, respectively, for YDFB, NCs, ALKs, and gelatin as follows. The large band of Amide A (3500–3000 cm^−1^), from N-H stretching linked to intermolecular H-bonds, was recorded at 3280/3239/3284/3266 cm^−1^. Amide B, corresponding to asymmetric stretching of +NH3 and =C-H, presents peaks at 2923/2970/2922/2921 cm^−1^. Amide I (1650–1600 cm^−1^), related to carbonyl group stretching coupled to a carboxyl group [45], is represented by the peaks at 1628/1643/-/1628 cm^−1^, suggesting a random structure for YDFB, gelatin, and NCs [46], and a loss of structure for ALKs. Amide II peaks (1550–1400 cm^−1^) are caused by deformation of N-H bonds [47] and COO^-^ stretching; they stand at 1402/1401/1406/1529 cm^−1^. Amide III, with C-N stretching vibrations coupled to N-H bending, reflecting the triple helix structure [8], is found at 1236/1230/1230/1234 cm^−1^.

#### 2.2.4. Color Analysis

Color is considered one of the most important parameters for the consumers in food acceptability. The results of the distribution in the CIELab color space, reported in Table 6, indicate a light, yellow color for NCs and a bright yellow-reddish color for ALKs, probably linked to the presence of lipids. As regards gelatin, commercial varieties have a range of colors in solution that go from a very pale yellow to a dark amber [48]. The color of the gelatin depends on the starting sources (i.e., different animals and different part of the same animal) and the treatments involved in the extraction process, such as the drying method [49]. Compared to mammalian gelatin, marine gelatins were found to have a more yellowish color due to higher content in cysteine and methionine [50]. The obtained results (L* = 314.95, a* = 1.32, b* = 40.02) were compared with those reported by Pranoto et al. [51] (L* = 60.37, a* = 13.67, b* = 35.57) from dried skin of yellowfin tuna, indicating that following this flowchart and starting with dehydrated mixed unsorted side streams of yellowfin tuna allows one to achieve a brighter gelatin that is less red and more yellow (light amber). Furthermore, in this research, the color attributes could be mainly linked to the drying process; in fact, oven-dried gelatin visually appeared much more colored than spray-dried and freeze-dried ones (see Tables S1 and S2 in the Supplementary material).

### 2.3. Additional Analyses on Extracted Gelatin

#### 2.3.1. pH

The pH value of gelatin obtained from YDFB, made up of raw unsorted yellowfin tuna side streams, mostly constituted by bones and connective tissue, was 3.63 ± 0.01, comparable with the one reported by Biluca et al. for skin (3.20) and bones (2.98) [52]. The pH value is strongly influenced by the chemical treatment [37] and by the starting material. Nurimala et al. [42] reported a value of 4.94 for yellowfin tuna skin, similar to that obtained from the same starting raw material by Rahman et al. (5.46–5.69) [28].

#### 2.3.2. Rheological Analyses

**Figure 6:**
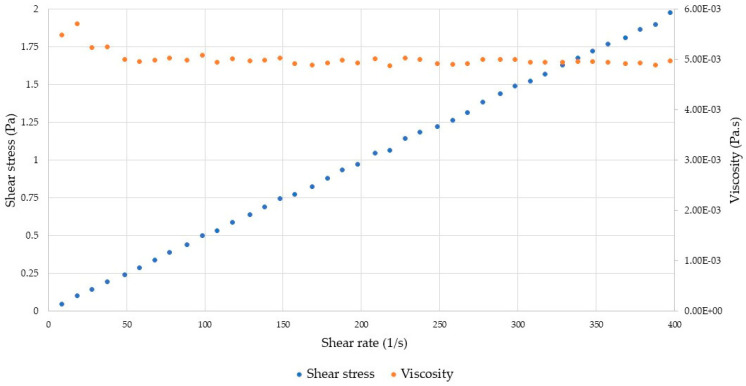
Viscosity of extracted gelatin.

The viscosity of gelatin extracted from unsorted mixed raw tuna by-products was 5.0 mPa·s, showing a Newtonian-like behavior (Figure 6), since the viscosity is almost constant regardless of the applied stress. Mafazah et al. [53] have reported a viscosity value equal to 8.6 mPa·s from yellowfin tuna skin gelatin, while higher values were recorded by Jeya Shakila et al. [54] from red snapper bones (15.30 mPa·s) and from grouper bones (18.50 mPa·s). Mahmood et al. stated that commercial gelatins have a viscosity that stands between 2–7 mPa·s [17], while higher values are needed for special applications (i.e., pharmaceutical, over 25 mPa·s; photography, over 78 mPa·s) [33]. However, the viscosity of a gelatin solution can be modified by changing the concentration of gelatin or by including additives to the formulation according to the specific interest and desired application.

**Figure 7:**
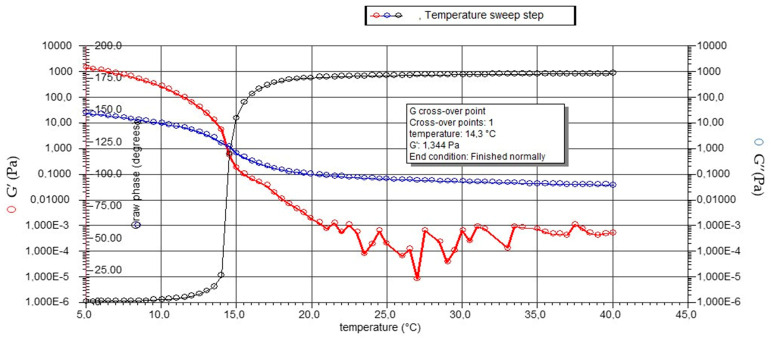
DVB of extracted gelatin. The G′ (elastic or storage modulus, red line) and G′′ (viscous or loss modulus, blue line) values as a function of temperature are reported together with the raw phase (black line).

The DVB (Dynamic Viscous-elastic Behavior) shows the effect of temperature, recorded in a dynamic temperature ramp mode from 5 to 40 °C, on the gelatin solution. The cross-over point between the elastic/storage modulus (G’, red line) and the viscous/loss modulus (G’’, blue line) during the cooling, from 40 to 5 °C, represents the gelling point, which is equal to 14.3 °C (Figure 7). This value is lower than the 18.7 °C achieved by Cho et al. [55] for the gelatin obtained from yellowfin tuna skins. However, the gelling temperatures of gelatins obtained from other fish species’ skin are usually lower: the maximum gelling temperature achieved with gelatin from Dover sole skins was 13 °C [56] and from megrim skins was 12 °C [57].

## 3. Conclusions

During the last few years, fishery production has significantly increased, and consequently the disposal of the so generated side streams has become an urgent environmental and economic concern to overcome. One of the EcoeFISHent project’s main goals is to deploy sustainable and efficient uses of fish-processing side streams by extracting bioactive compounds for high-value-added food supplements and skin care products, as well as biodegradable and compostable barrier layers for food packaging, feed, and fertilizers/plant bio-stimulants.

The traditional methods for the extraction of gelatin start from separated by-products, such as skin, scales, and bones. This step is economically unaffordable for many stakeholders which transform fishes like tuna for canning. Nevertheless, this kind of industries generates a lot of by-products that remains unexploited for this economic reason. In this work, the authors underline the importance of the first extractive part from non-separated biomass which treats real industrial sample volumes at an industrial scale of the order of 16,000 tons per year. The most challenging characteristic is precisely its high complexity that differentiates it from the usual fresh or frozen selected biomass already described in several articles. In detail, the possibility of starting from unsorted side-streams is very promising because the sorting step to separate the different fractions such as bones, fins, heads, and skins is too expensive and time-consuming for many industrial realities, such as those considered in this paper. One of the major problems for the valorization of the side streams of the fish supply chain is precisely the treatment of non-separated biomass like fish filleting processing residues but also by-catches and/or unwanted catches (undersized fish). This is precisely one of the main objectives of the Horizon 2020 EcoeFISHent project as cited https://ecoefishent.eu/the-six-circular-value-chains/ “At the core of the EcoeFISHent project there is a systemic solution, related to Fish Processing Side-stream, based on implementation of the unique EcoeFISHent pre-processing Pilot Unit (EPPU) for unsorted residues processing. EPPU aims to obtain the so-called Eco-FISH-powder (EFP), a dry concentrated bioactive powder to be further purified and employed in different products integrations”(accessed on 1 September 2023).

For this reason, the authors paid much attention to the optimization of the pre-treatment and the extraction steps (Figure 2) starting from samples of about 1–1.5 kg of dried biomass derived from 150 kg of fresh industrial by-products previously dehydrated by a patented industrial process. All the cited steps, even those necessarily at the lab scale, are managing large amounts (1.5 kg) of samples (differently from the majority of published papers which treat an average of 100–500 g of biomass [58,59]) using reagents (e.g., industrial enzymes) and a limited number of steps (e.g., limited numbers of sieving and centrifugations), saving as much as energy as possible.

The second novelty of this paper is related to the managing of dehydrated starting biomass, which is a crucial novelty of the H2020 EcoeFISHent project, since it makes possible the logistics of this huge amount of by-products. To monitor the impact of this dehydration step, both the TBARS assay and the peroxide test were chosen to check the oxidation. Moreover, some microbiological analyses were reported (sulfite-reducing clostridia and spores, TVC, coliforms, Escherichia coli β-gluc. +, Enterobacteriaceae, Staphylococci c. + at 37 °C, Salmonella spp., Listeria monocytogenes, and histamine) to confirm the completed biomass stabilization after the patented industrial dehydration treatment by Themis S.p.A.

In particular, gelatin extracted from this kind of raw tuna side stream has shown to have specific viscoelastic properties, similar to those reported in the literature for non-dehydrated and previously separated fish leftovers (i.e., tuna skin), and further investigations for its application are currently in fieri. Moreover, it should be emphasized that in the same extraction process it is also possible to obtain NCs (Non-Collagenous proteins), with a good content of nutritionally valued proteins exploitable in dietary products and high-protein foods, and above all gelatin hydrolysates (HGPs) as a further potentially bioactive product, the characterization of which will be the subject of a future paper (Part II).

## 4. Materials and Methods

### 4.1. Samples and Chemicals

**Figure 8:**
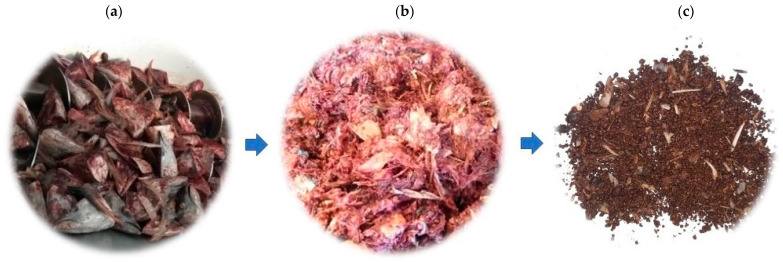
Biomass pre and post industrial dehydration: (**a**) fish biomass constituted by tuna side streams; (**b**) NDFB (Not Dehydrated Fish Biomass); (**c**) YDFB (Yes Dehydrated Fish Biomass).

Samples of fish biomass (NDFB and YDFB) were derived from unsorted, mixed canned yellowfin tuna processing side streams of Generale Conserve (ASdoMAR^®^). The canned product is composed of the fillet of tuna, thus generating a big quantity of side stream material made up of raw (crude) heads, fins, bones, skins, and tails. In order to valorize this less noble biomass, it is treated with a process patented by Themis S.p.A. [60] which allows one to obtain a fish powder (YDFB, Figure 8) with low residual moisture (Table 1). YDFB represents a type of industrial sample derived from different batches, each one obtained by dehydrating 150 kg of raw materials which enables a reduction of about 2/3 of the initial weight of the biomass, produced during the first 18 months of EcoeFISHent project.

Ultrapure Milli-Q water (18 MΩ), generated by a Millipore Milli-Q system (Bedford, MA, USA) was used throughout the course of experiments; moreover, all chemicals and reagents were of analytical grade. Enzyme 3G PBN-66 L, as proteolytic serine hydrolase (from fermentation of *Bacillus licheniformis*), supplied by 3G SINCE 2000 (Barcelona, Spain), was used in its optimal conditions of temperature (60 °C) and pH (9.0–10.0). Sodium hydroxide, hydrochloric acid, citric acid, acetic acid, diethyl ether, thiobarbituric acid, trichloroacetic acid, 1-butanol, and isopropanol were provided by Sigma-Aldrich Chemical Company (Steinheim, Germany).

### 4.2. Preliminary Analyses

#### 4.2.1. Proximate Composition and Monitoring of Lipid Oxidation of Samples Pre (NDFB) and Post (YDFB) Industrial Dehydration

Samples, after a preliminary homogenization step, were analyzed in duplicate following official methods in order to characterize them in terms of residual moisture (AOAC 950.46B) [61], protein (AOAC 981.10) [61], ashes (AOAC 942.05) [61] and lipid [62] contents.

A TBARS (Thiobarbituric-Acid-Reactive Substances) test was performed as reported by Hu et al. [63] directly on the samples to evaluate the secondary oxidation products. The TBARS solution was prepared with 15 g of trichloroacetic acid and 0.75 g of thiobarbituric acid dissolved in 100 mL of a mixture composed of 40 mL of 1-butanol, 40 mL of isopropanol, and 20 mL of 0.5 M HCl (in water). Samples of YDFB were weighed in Pyrex tubes and the TBARS solution was added, then the samples were incubated at 95 °C for 2 h in a water thermostatic bath (E200, LAUDA, Germany). The tubes were then cooled, and samples were read using a UV–Visible spectrophotometer (Agilent Cary 100 Varian Co., Santa Clara, CA, USA) at 532 nm after obtaining a calibration curve with MDA (1,1,3,3-tetramethoxypropane) as the standard in a range of 0.61–6.08 μM.

The Peroxide Value (PV) test, as an additional analysis for the monitoring of lipid oxidation, was carried out following the official method ISO 5.4.205 rev.1 2020.

#### 4.2.2. Microbiological Analyses

In this study, microbiological analysis was conducted to assess the efficiency of the patented industrial dehydration treatment in stabilizing such a perishable matrix. Sulfite-reducing clostridia content was determined using the official method of ISO 15213:2003. Total Viable Count (TVC) was evaluated according to the method of ISO 4833-1:2013. Coliforms (ISO 4832:2006), Escherichia coli β-gluc. + (ISO 16649-2:2001), Enterobacteriaceae (ISO 21528-2:2017), Staphylococci c. + at 37 °C (NF V 08-057-1 2004), Salmonella spp. (UNI EN ISO 6579-1:2020), and Listeria monocytogenes (UNI EN ISO 11290-1:2017) were subjected to microbiological characterization according to the reported methods. Histamine has been evaluated according to the method of IS 5.4.136 rev.2 2020, whereas the Acid–Base Volatile Total (ABVT) was evaluated according to the provisions of the Regulation CE 2074/2005 05/12/2005 GU CE L338 22/12/2005 All I sez II cap III-IV + Reg UE 627/2019 15/03/2019 GU UE L131 -17/05/2019 All VI Capo II.

### 4.3. Enzymatic Extraction of Proteins

The extraction process can be divided in three principal steps: pre-treatment, extraction, and drying; after pre-treatment, NCs and ALKs were extracted, and after gelatin extraction HGPs were obtained from the residual solid (RS) by an enzymatic hydrolysis and a drying step.

Figure 2 resumes the flowchart of the whole process, and a detailed description is reported in the following paragraphs: pre-treatment (Section 4.3.1); gelatin and Hydrolyzed Gelatin Peptides (HGPs) extraction (Section 4.3.2).

#### 4.3.1. Pre-Treatment

Removal and recovery of Non-Collagenous proteins (NCs) and Alkali Hydrolysates (ALKs): The initial enzymatic hydrolysis was performed as follows. A total of 1–1.5 kg of dried samples (YDFB) were weighed in Pyrex glass bottles, water 1/6 (*w*/*v*) was added, and pH was adjusted to 9.0 before the addition of enzyme 3G PBN66L (from fermentation of *Bacillus licheniformis*) 1% (*w*/*v*). The reaction was carried out at 60 °C, representing the optimal temperature of incubation for this kind of enzyme, for 2 h with continuous stirring in a shaking incubator (SKI 8R, Argo Lab, Kunshan, China); after that, the enzyme was inactivated at 90 °C for 15 min in a water thermostatic bath (E200, LAUDA, Germany). The solution was centrifuged at 1150× *g* for 15 min (Refrigerated Benchtop Centrifuge 5810/5810 R, Eppendorf, Hamburg, Germany); the supernatant was filtered with filter paper before spray-drying and NCs powder was obtained.

To remove lipids and the remaining non-collagenous proteins [53], an alkali treatment was performed by adding to the solid residue a solution of NaOH 0.2 M (1/4 *w*/*v*) with continuous stirring for 30 min at room temperature; therefore, a centrifugation at 1150× *g* for 15 min was followed, and the supernatant was collected to spray-dry and characterize the correspondent powder (ALKs). Both NCs and ALKs were characterized in terms of proximate analysis, aminoacidic composition, FT-IR spectra, and color.

Demineralization and Fibers Swelling:

The solid residue remaining after the pretreatment step, was washed with water by sieving it (Ø 200 μm) to obtain a biomass mainly composed of bones and fish scales, which are the main sources of gelatin, and a demineralization was carried out with HCl 1 M (1/10 *w*/*v*) for 4 h at room temperature with constant stirring. After filtration, the solid was washed with water and then a solution of citric acid 0.05 M (1/6 *w*/*v*), for fiber swelling and facilitation of gelatin extraction while removing fish odor, was added, and continuously stirred for 2 h at room temperature.

#### 4.3.2. Gelatin and Hydrolyzed Gelatin Peptides (HGPs) Extraction

After filtration and washing with water, the extraction was carried out in water (1/5 *w*/*v*) for 16 h at 60 °C by gently stirring in a shaking incubator; then, the solution was filtered with filter paper and oven-dried at 60 °C for 8 h in convective mode/spray-dried or lyophilized (Freeze-dryer Büchi Lyovapor L200I S, Büchi Labortechnik AG, Flawil, Switzerland) (Figure 4a–c).

The remaining solid (RS) was used to extract HGPs by enzymatic hydrolysis [64]. Water (1/3 *w*/*v*) was added to the leftover solid, pH was adjusted until reaching a value of 9, and proteolytic enzyme 3G PBN66L 0.2% (*w*/*v*) was included. The enzymatic hydrolysis was performed for 2 h at 60 °C with continuous stirring and the last enzymatic inactivation was followed as in the first step (i.e., 90 °C, 15 min). Then, the solution was filtered with filter paper and the filtrate was oven-dried at 60 °C for 8 h in convective mode/spray-dried or lyophilized. A preliminary study of the electrophoretic profile was conducted (data not shown); nevertheless, results need to be confirmed and detailed by a more accurate and precise technique such as chromatographic analysis (GPC, Gel Permeation Chromatography). The results of the molecular weight distribution assays will be discussed in Part II since they are strictly correlated with the biological activities of the extracted compounds.

### 4.4. Yields

The yields of NCs, ALKs, gelatin, and HGPs were calculated as reported below.

(1) $$Yield\left(\%\right)=\frac{weight\;of\;dried\;extract\;\left(g\right)}{weight\;of\;starting\;material\;(g)}\times 100$$

### 4.5. Characterization of Protein Extracts

After the extraction process, a characterization of the so-obtained bioactive compounds was performed as follows. As far as the HGPs are concerned, their characterization is currently in fieri, and it will be the object of Part II.

#### 4.5.1. Proximate Analysis

Extracted protein hydrolysates were analyzed following the AOAC Official Method (2005). Residual moisture was determined in an oven at 105 °C, the Kjeldahl method was performed for the identification of crude proteins using 6.25 as the conversion factor, ashes were gravimetrically calculated after calcination of the organic matter, and lipids were extracted with diethyl ether and measured by gravimetry.

#### 4.5.2. Amino Acids Analysis

The analysis of the amino acid composition was performed following the protein hydrolysis method proposed by Lorenzo et al. [65]. After the hydrolysis, samples were derivatized applying the protocol of Domínguez et al. [66] and finally chromatographically analyzed.

#### 4.5.3. ATR-FTIR Analysis

A qualitative determination was performed by Attenuated Total Reflectance Fourier-Transform Infrared (ATR-FTIR) spectroscopy. Spectra were recorded with a FT-IR spectrophotometer (Perkin Elmer, Inc., Waltham, MA, USA) from 4000 to 600 cm^−1^ at room temperature with a resolution of 4 cm^−1^ and accumulating 8 scans per sample; a background was recorded before measurements.

#### 4.5.4. Color Analysis

The color was measured in the range between 300 and 900 nm using a double-beam UV–Visible spectrophotometer (Agilent Cary 100 Varian Co., Santa Clara, CA, USA) endowed with an integrating sphere (Varian DRA), able to disperse the light such that it covers every inside surface equally, with a resolution of 1 nm. A white Spectralon^®^ disk was used as reference and analyses were performed in duplicate. The CIELab coordinates L* (lightness), a* (greenish-reddish), and b* (bluish-yellowish) were automatically computed by the software Cary100 Color from the raw spectral data using the CIE D65 illuminant.

### 4.6. Additional Analyses on Extracted Gelatin

#### 4.6.1. pH

The pH was measured following the British Standard Institution method (1975).

A solution of 1% gelatin (g/mL) was prepared by dissolving gelatin in water at 60 °C for 30 min, and the pH was measured at room temperature (25 ± 1 °C) with a pH meter (HI5221, Hanna Instruments, Villafranca Padovana, Padova, Italy) after performing calibration.

#### 4.6.2. Rheological Analyses

The rheological and viscoelastic properties of the extracted gelatin were determined with a rheometer (AR1000 Advanced Rheometer, TA Instruments, West Sussex, UK) as follows: a solution of 6.67% (*w*/*v*) of gelatin in water was prepared by heating it at 60 °C for 30 min and then cooled at room temperature [43] before being analyzed. As far as the viscosity is concerned, a 40 mm 2° Al cone geometry was used, with 64 μm of physical gap at a controlled temperature of 40 °C with a shear rate from 0 to 400 s^−1^. Viscosity was calculated as the slope in the representation of the shear rate (in x axis), and the shear stress (in y axis) with a Newtonian behavior.

The gelling point determination was carried out with 40 mm 2° Al cone geometry in a temperature range of 5–40 °C, with a temperature increment equal to 0.5 °C. An oscillatory stress of 3.0 Pa was applied with a frequency of 1 Hz. The elastic modulus (G’) and the viscous modulus (G’’) were monitored with the temperature according to those reported by Gómez-Guillén et al. [67]. The cross-over point between the modules (G’ and G″) during the cooling (from 40 to 5 °C) corresponds to the gel point.

### 4.7. Statistical Analysis

All analyses were carried out in duplicate, unless otherwise was stated, and the results were reported as average value ± standard error. Data were analyzed using the Excel Data Analysis Tool (Microsoft Corporation, Seattle, WA, USA). Analysis of variance (ANOVA), at a significance level of 0.05, was performed to analyze the mean significant differences among the samples.

## Figures and Tables

**Table 1:** Proximate analysis of samples, both pre (NDFB) and post (YDFB) industrial dehydration.

Analyses	Pre Dehydration (NDFB): Results ^1^ (g/100 g)	Post Dehydration (YDFB): Results ^1^ (g/100 g)
Residual moisture	62.2 ± 0.1	4.7 ± 0.1
Crude proteins	19.5 ± 0.4	45.9 ± 1.7
Lipids	5.1 ± 0.1	13.0 ± 0.5
Ashes	13.2 ± 0.1	32.5 ± 0.8

^1^ Results are expressed as mean value ± standard error.

**Table 2:** Microbiological analyses of samples *pre* (NDFB) and *post* (YDFB) industrial dehydration.

	NDFB (Not Dehydrated Fish Biomass)	YDFB (Yes Dehydrated Fish Biomass)
Sulfite-reducing clostridia and spores (CFU/g)	<10	<10
Total viable count (CFU/g)	2300	3300
Coliforms (CFU/g)	<10	<10
Escherichia coli β-gluc. + (CFU/g)	<10	<10
Enterobacteriaceae (CFU/g)	<10	<10
Staphylococci c. + at 37 °C (CFU/g)	<10	<10
Salmonella spp. (CFU/g)	Absent	Absent
Listeria monocytogenes (CFU/g)	Absent	Absent
Histamine (mg/kg)	<5	<5
Acid–Base Volatile Total Nitrogen (mg/100 g)	-	14.9

**Table 3:** Proximate analyses and yields of extracted NCs (Non-Collagenous proteins) and ALKs (Alkali Hydrolysates).

Analyses	NCs Results ^1^ (g/100 g)	ALKs Results ^1^ (g/100 g)
Yield	14.6	18.3
Residual moisture	5.3 ± 0.2	5.9 ± 0.1
Crude proteins	67.0 ± 5.0	33.0 ± 2.0
Lipids	0.2 ± 1.8	27.5 ± 1.6
Ashes	24.0 ± 2.0	16.8 ± 1.5

^1^ Results are expressed as mean value and mean value ± standard error.

**Table 4:** Proximate analysis of extracted gelatin.

Analyses	Results ^1^ (g/100 g)
Residual moisture	2.0 ± 0.2
Crude proteins	88.0 ± 2.9
Lipids	0.2 ± 0.0
Ashes	5.1 ± 0.4

^1^ Results are expressed as mean value ± standard error.

**Table 5:** Aminoacidic composition of the starting material YDFB (Yes Dehydrated Fish Biomass), and obtained extracts: NCs (Non-Collagenous proteins), ALKs (Alkali Hydrolysates), and gelatin.

YDFB			NCs			ALKs			GELATIN		
AA	Results ^1^ (g/100 g of YDFB)	Results ^1^ % AA	AA	Results ^1^ (g/100 g of NCs)	Results ^1^ % AA	AA	Results ^1^ (g/100 g of ALKs)	Results ^1^ % AA	AA	Results ^1^ (g/100g of GELATIN)	Results ^1^ % AA
Gly	7.51 + 0.80	13.80	Gly	10.28 + 0.47	14.59	Glu	2.42 + 0.01	10.34	Gly	21.23 + 2.26	24.57
Glu	6.49 + 0.49	11.92	Glu	8.58 + 0.29	12.18	Asp	2.26 + 0.02	9.66	Arg	8.30 + 0.95	9.61
Asp	4.60 + 0.39	8.45	Asp	5.64 + 0.15	8.00	Leu	2.12 + 0.03	9.05	Glu	8.27 + 0.85	9.57
Ala	4.21 + 0.43	7.73	Ala	5.55 + 0.22	7.87	Gly	1.79 + 0.01	7.67	Pro	8.22 + 0.84	9.51
Lys	3.73 + 0.42	6.86	Arg	5.11 + 0.33	7.25	Ala	1.65 + 0.03	7.07	OH-Pro	8.07 + 0.77	9.34
Arg	3.67 + 0.65	6.75	Pro	4.81+ 0.20	6.82	Val	1.53 + 0.01	6.56	Ala	7.96 + 0.77	9.22
Pro	3.62 + 0.30	6.65	Lys	4.32 + 0.08	6.14	Phe	1.39 + 0.02	5.95	Asp	4.42 + 0.40	5.12
Leu	3.47 + 0.31	6.37	Leu	4.05 + 0.21	5.74	Pro	1.35 + 0.01	5.79	Lys	3.43 + 0.37	3.97
Val	2.49 + 0.22	4.58	Ser	3.18 + 0.19	4.52	Ile	1.33 + 0.01	5.71	Ser	3.27 + 0.36	3.78
Ser	2.46 + 0.20	4.52	OH-Pro	3.05 + 0.19	4.32	Arg	1.33 + 0.04	5.70	Thr	2.67 + 0.23	3.09
OH-Pro	2.13 + 0.12	3.92	Val	2.94 + 0.15	4.17	Ser	1.09 + 0.06	4.65	Leu	2.28 + 0.25	2.64
Ile	1.96 + 0.18	3.61	Thr	2.92 + 0.17	4.14	Lys	1.08 + 0.08	4.62	Val	2.02 + 0.22	2.34
Phe	1.82 + 0.09	3.34	Ile	2.18 + 0.12	3.10	Thr	1.03 + 0.01	4.43	Phe	1.81 + 0.20	2.10
Hys	1.47 + 0.09	2.69	Phe	1.90 + 0.10	2.69	Tyr	1.01 + 0.04	4.32	Met	1.49 + 0.14	1.73
Met	1.40 + 0.09	2.57	Met	1.79 + 0.12	2.53	Hys	0.69 + 0.04	2.95	Hys	1.32 + 0.18	1.53
Thr	1.29 + 0.06	2.38	Hys	1.74 + 0.11	2.47	Met	0.67 + 0.04	2.87	Ile	1.06 + 0.12	1.23
Tyr	1.13 + 0.05	2.07	Tau	1.08 + 0.07	1.53	OH-Pro	0.41 + 0.02	1.77	Tyr	0.42 + 0.05	0.49
Tau	0.51 + 0.02	0.93	Tyr	1.01 + 0.07	1.43	Tau	0.11 + 0.01	0.48	Cys	0.14 + 0.05	0.16
Cys	0.47 + 0.04	0.86	Cys	0.35 + 0.03	0.49	Cys	0.10 + 0.01	0.44	Tau	0.00 + 0.00	0.00

^1^ Results are expressed as mean value ± standard deviation.

**Table 6:** CIELab parameters of extracted NCs (Non-Collagenous proteins), ALKs (Alkali Hydrolysates) and Gelatin.

	NCs	ALKs	Gelatin
**CIELab**	L* = 297.97 ± 0.03	L* = 357.05 ± 0.02	L* = 314.95 ± 0.03
	a* = −3.38 ± 0.001	a* = 8.02 ± 0.01	a* = 1.32 ± 0.001
	b* = 16.77 ± 0.01	b* = 70.75 ± 0.02	b* = 40.02 ± 0.00

## Data Availability

Data is contained within the article.

## Supplementary Materials

The following supporting information can be downloaded at: https://www.mdpi.com/article/10.3390/gels9090760/s1, Figure S1: FTIR Spray-dried and Freeze-dried gelatins. Table S1: Color parameter of Spray-dried gelatin and Freeze-dried gelatin. Table S2: Color parameter of NCs, ALKs, and Oven-dried gelatin.
