# Supplementary material for: Feasibility of Enzymatic Protein Extraction from a Dehydrated Fish Biomass Obtained from Unsorted Canned Yellowfin Tuna Side Streams: Part I
Source: Gels. 2023 Sep 18;9(9):760. doi: 10.3390/gels9090760 (PMC10531079; doi:10.3390/gels9090760)
Supplement: Supplementary file 1 [file gels-09-00760-s001.zip › Table S1.pdf]

Table S1: Color parameter of Spray-dried gelatin and Freeze-dried gelatin.

	**Spray-dried Gelatin**	**Freeze-dried Gelatin**
**CIELab**	L* = 472.2671<br>a* = -1.6761<br>b* = 19.3046	L* = 371.0127<br>a* = 0.2594<br>b* = 43.6726
**Tristimulus**	X = 7071.2491<br>Y = 7457.5894<br>Z = 7574.1517	X = 3531.3445<br>Y = 3713.6568<br>Z = 3300.4208
**Whiteness**	WI = 7437.5281	WI = 3656.5520
**Tint Index**	T = 7455.8278	T = 3706.5397
**Yellowness ASTM E313-00**	YI = 8.0015	YI = 22.7383
**Gardner ASTM D6166**	Gtm = 1.8533	Gtm = 3.3191
**Gardner DIN EN1557**	Gtm = 1.8505	Gtm = 3.3199
