# Supplementary material for: Feasibility of Enzymatic Protein Extraction from a Dehydrated Fish Biomass Obtained from Unsorted Canned Yellowfin Tuna Side Streams: Part I
Source: Gels. 2023 Sep 18;9(9):760. doi: 10.3390/gels9090760 (PMC10531079; doi:10.3390/gels9090760)
Supplement: Supplementary file 1 [file gels-09-00760-s001.zip › Table S2.pdf]

Table S2: Color parameter of NCs, ALKs and Oven-dried gelatin.

	NCs	ALKs	Gelatin
**Tristimulus**	X = 1870.4936<br>Y = 1982.7871<br>Z = 1964.3733	X = 3208.7604<br>Y = 3325.9503<br>Z = 2553.0068	X = 2213.4009<br>Y = 2322.3236<br>Z = 2033.0548
**Whiteness**	WI = 1955.6327	WI= 3230.7691	WI = 2261.2001
**Tint Index**	T = 1981.6514	T= 3309.6017	T = 2314.0167
**Yellowness ASTM E313-00**	YI = 10.1987	YI= 38.2670	YI = 24.5286
**Gardner ASTM D6166**	Gtm = 2.3176	Gtm= 4.2726	Gtm = 3.4203
**Gardner DIN EN1557**	Gtm = 2.2618	Gtm= 4.3417	Gtm = 3.4353
