# Supplementary figures and images for: Feasibility of Enzymatic Protein Extraction from a Dehydrated Fish Biomass Obtained from Unsorted Canned Yellowfin Tuna Side Streams: Part I
Source: Gels. 2023 Sep 18;9(9):760. doi: 10.3390/gels9090760 (PMC10531079; doi:10.3390/gels9090760)

**Figure S1:** FTIR Spray-dried and Freeze-dried gelatins.

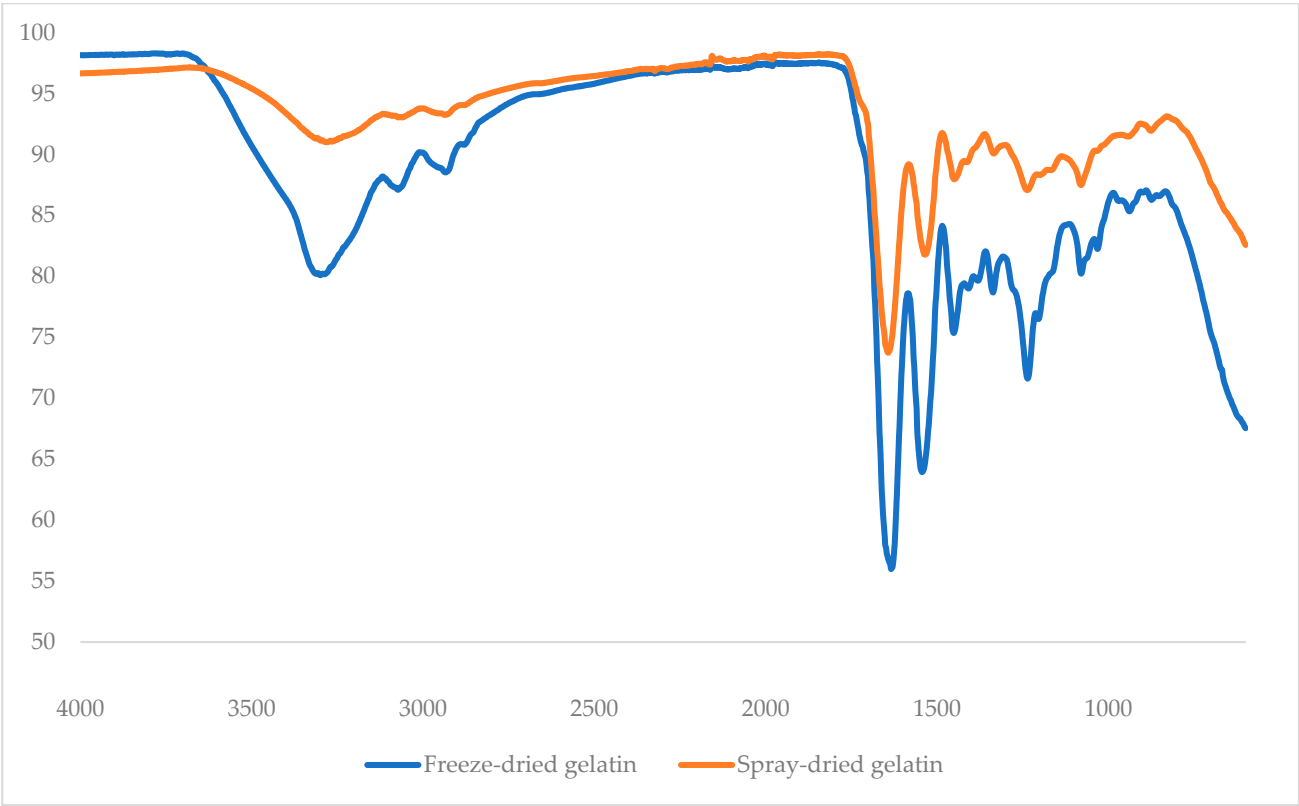

Supplement: Supplementary file 1 [file gels-09-00760-s001.zip › Figure S1.pdf]

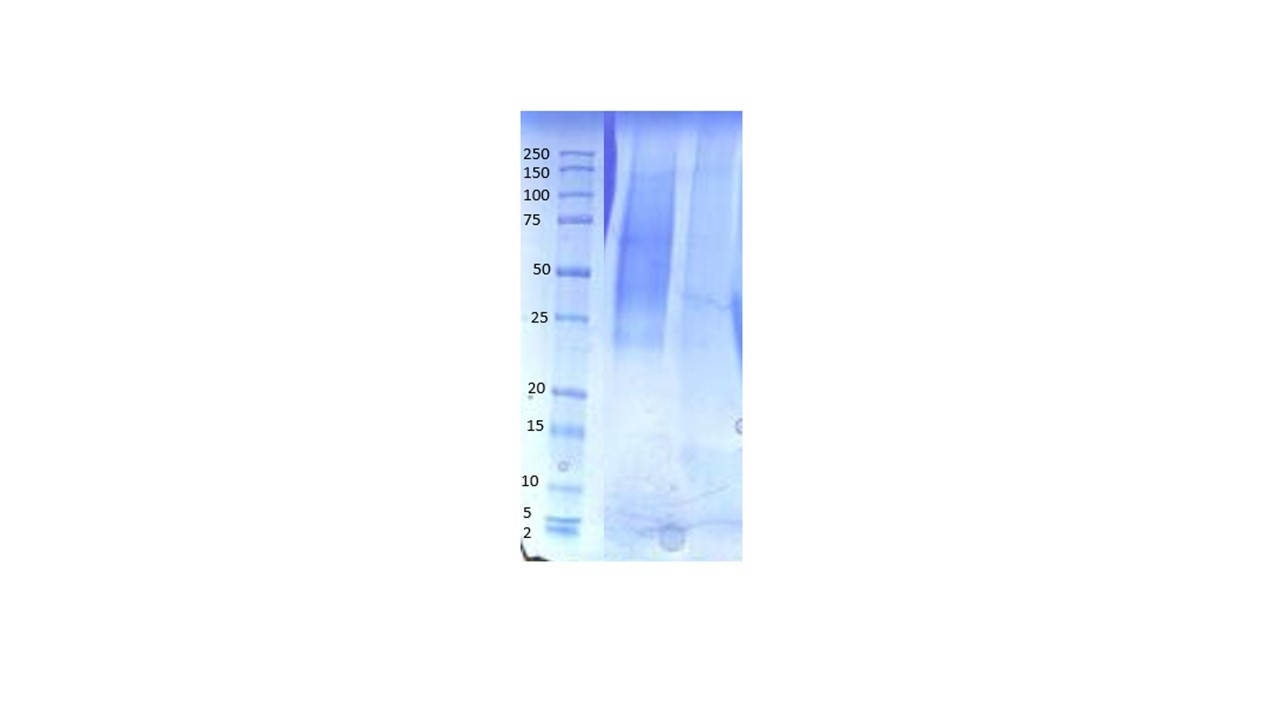

Supplement: Supplementary file 1 [file gels-09-00760-s001.zip › Figure S2.jpg]
